# Effects of Sublethal Concentrations of Cyantraniliprole on the Development, Fecundity and Nutritional Physiology of the Black Cutworm *Agrotis ipsilon* (Lepidoptera: Noctuidae)

**DOI:** 10.1371/journal.pone.0156555

**Published:** 2016-06-01

**Authors:** Chunmei Xu, Zhengqun Zhang, Kaidi Cui, Yunhe Zhao, Jingkun Han, Feng Liu, Wei Mu

**Affiliations:** 1 College of Plant Protection, Shandong Provincial Key Laboratory for Biology of Vegetable Diseases and Insect Pests, Shandong Agricultural University, Tai’an, Shandong 271018, P.R. China; 2 College of Horticultural Science and Engineering, Shandong Agricultural University, Tai'an, Shandong 271018, PR China; University of Basilicata, ITALY

## Abstract

To better understand the sublethal effects of cyantraniliprole on the black cutworm *Agrotis ipsilon* (Lepidoptera: Noctuidae), several studies were carried out to investigate sublethal effects on development stages, population parameters, feeding indices and nutrient content of *A*. *ipsilon*. The result of a bioassay showed that cyantraniliprole had high toxicity against *A*. *ipsilon* fourth-instar larvae with an LC_50_ of 0.354 μg.g^−1^ using an artificial diet. Compared with controls, sublethal doses of cyantraniliprole at LC_5_, LC_20_ and LC_40_ levels prolonged larval and pupal duration and extended mean generation time and total preovipositional period. In addition, survival rate, reproductive value, intrinsic and finite rates of increase and net reproduction rate declined significantly. Meanwhile, cyantraniliprole had markedly antifeedant effects; decreased the relative growth rate (RGR), the relative consumption rate (RCR), the efficiency of conversion of ingested food (ECI), the efficiency of conversion of digested food (ECD); and increased the approximate digestibility (AD) significantly. This phenomenon contributed to the decrease of nutrient contents, including lipids, protein and carbohydrates, to the point that insufficient energy was available for normal growth. Therefore, sublethal concentrations of cyantraniliprole decreased growth speed and reduced population reproduction of *A*. *ipsilon*. This result provides information useful in integrated pest management (IPM) programs for *A*. *ipsilon*.

## Introduction

The black cutworm *Agrotis ipsilon* (Lepidoptera: Noctuidae) is a polyphagous pest that occurs worldwide and can destroy more than one hundred types of crops [1]. *A*. *ipsilon* larvae always cause crop deficiency and broken ridge in unprotected crop fields by cutting off seedlings or tunneling into the bases of older plants and destroying their growing points, which can cause huge economic losses [2,3]. Chemical control is currently a standard practice for managing *A*. *ipsilon* because non-chemical control measures alone usually do not adequately prevent economic damage. However, *A*. *ipsilon* has developed an increased resistance to organophosphates, carbamates and pyrethroids due to high-frequency applications and exposure to high concentrations of these traditional chemical pesticides [2]. Currently, new insecticides with unique modes of action are required as an alternative for an integrated *A*. *ipsilon* management program.

Cyantraniliprole is a novel anthranilic diamide insecticide. It targets the ryanodine receptors in insect muscles and affects control of calcium channels [4,5]. Cyantraniliprole’s adverse effects on insects, such as muscle cramps and feeding cessation, occur via activation of ryanodine receptors and disruption of calcium balances [5,6]. In addition, anthranilic diamide insecticides also exhibit a good selectivity to non-target arthropods and outstanding mammalian safety, along with exceptional safety on avian and aquatic organisms[5], thus making it very suitable for use in integrated pest management (IPM) programs. Many previous studies showed that cyantraniliprole had high insecticidal activity on several insect species such as lepidopteran [7–9], hemipteran [10] and dipteran [11–13]. Du et al. also showed that cyantraniliprole has shown high insecticidal activity against *A*. *ipsilon* third instar larvae [3].

When target pests are not killed immediately after the application of insecticide in the field, sublethal effects, such as physiological and behavioral changes, could appear as the dose of insecticide is reduced over time [14]. Therefore, sublethal doses of insecticides could have a large influence on insect emergence rate, sex ratio, pupal weight, adult reproduction and the duration of larvae and pupae [15].

Insecticides with different modes of action or application methods produce various effects against targeted insect pests at sublethal doses. For example, some insecticides stimulate the reproductive capacities of target pests at low concentrations [11,16], while other studies indicated that adult fecundity and survival rates would decrease with sublethal doses of insecticides [14,15]. These contrasting effects reported in literature indicate that studying sublethal effects of insecticides on targeted insect pests is crucial for guiding the scientific use of new pesticides, delaying the development of resistance and reducing the risk of pest resurgence.

However, sublethal effects of cyantraniliprole on *A*. *ipsilon* have not been reported. The objective of this study was to evaluate the possible sublethal effects of cyantraniliprole on *A*. *ipsilon* developmental and population parameters using the age-stage, two-sex life table. In this study, we also investigated the effects of cyantraniliprole on feeding indices and the accumulation of nutrient contents in *A*. *ipsilon* fourth-instar larvae. The results of this study will provide a scientific basis for reasonable use of this new type of insecticide in strategies for controlling *A*. *ipsilon* infestations.

## Materials and Methods

### Ethics Statement

The local farmers permitted us to collect *Agrotis ipsilon* in their fields, and no permits were required to collect this common insect. The sampling process did not involve the regulated, endangered or protected species.

### Insect rearing

The *A*. *ipsilon* larvae used in this study have been reared in our laboratory since 2006. Adults were collected from the field at Langfang, Hebei province (116°38′07″-44′06″E/39°28′42″-32′54″N) of China each summer and introduced to the colony to prevent inbreeding effects. Newly hatched larvae were transferred into 12-well cell culture plates and given an artificial diet [17]. Larvae were reared alone, and then transferred into finger-shaped glass tubes (2.2 cm in diameter, 8 cm in height) at the last instar larval stage, so that larvae could molt normally into the pupal stage. All the pupae were selected and then paired (one female with one male) after all the individuals had emerged. Adult moths were given a 20% honey solution as food. All the rearing procedures for *A*. *ipsilon* were maintained at 25 ± 1°C and 75 ± 5% relative humidity (RH) with a photoperiod of 16:8 h (L:D).

### Toxicity of cyantraniliprole for *A*. *ipsilon*

The method for determining the toxicity of cyantraniliprole on fourth-instar larvae of *A*. *ipsilon* was carried out by mixing an artificial diet with insecticide. First, a preparatory test was required to find the effective dose ranges. Cyantraniliprole (19% SC provided by DuPont Crop Protection) was diluted into a series of concentrations with distilled water, and then added to the artificial diet and mixed thoroughly at 1.6, 0.8, 0.4, 0.2, 0.1, and 0.05 μg.g^-1^. A control treatment was mixed with distilled water.

Newly molted active fourth-instar larvae were selected and starved for 4 hours before the experiments. The artificial diet was cut into similar-size cubes (approximately 1.5 g per cube) and put into 12-well cell culture plates, respectively. The experiment included seven treatments performed in five replications with 24 larvae in each replication. Mortality was assessed after 72 h. Larvae were considered dead if they were unable to move normally when disturbed with a writing brush or if they exhibited extreme shrinkage compared to the control larvae.

### The sublethal effects of cyantraniliprole on the growth and population parameters of *A*. *ipsilon*

In the present study, life table theory was used to provide a comprehensive evaluation of the sublethal effects of cyantraniliprole on *A*. *ipsilon*. The age-stage, two-sex life table scientific theory was originally developed to estimate population growth [16,18]. First, males can also cause economic damage and contribute to fecundity of the population. In addition, developmental rates varied among individuals, so that there were overlapping phenomena between stages [19,20].

In this part of the study, approximately 1500 eggs all laid on the same day were selected and raised. The developmental times of all individuals were recorded. When most individuals had turned into fourth-instar larvae, 480 were selected for the life table study, using 120 fourth-instar larvae for each treatment and considering each individual as one replication. Based on the toxicity of cyantraniliprole on *A*. *ipsilon*, the 120 newly molted fourth-instar larvae in each group were fed with an artificial diet treated with LC_5_, LC_20_ or LC_40_ concentrations of cyantraniliprole. The control group was fed an artificial diet treated with distilled water. These individuals were numbered and named for the parental generation. After 72 h of treatment, all the live insects were transferred into finger-shaped glass tube (2.2 cm in diameter, 8 cm in height), and fed with a fresh artificial diet containing no cyantraniliprole. The developmental duration of each individual was recorded twice a day until they reached the pupal stage. The artificial diet was kept fresh during the experiment to ensure normal growth. All subsequent treatments were applied to 80 ~ 100 randomly selected and weighed 2-day old pupae. All the pupae were transferred individually to small plastic cups (7.0 cm in diameter, 3.5 cm in height) and placed in a climate incubator (25 ± 1°C, 75 ± 5% relative humidity (RH) until the adults emerged. All the adults in each treatment were sexed and paired (one female with one male), and then placed in an oviposition plastic container (9.3 cm in diameter, 6.5 cm in height) with a 20% honey solution as food. Eggs on the gauze were collected every day and a new gauze was put in its place. Eggs were counted at the second day with naked eye. The adult pairs were given fresh honey solution every day until they died. The longevity of adults, the preovipositional period and the number of eggs produced by each female adult was recorded. All the measurements were recorded twice each day. From each treatment approximately 500 eggs laid within 24 hours were randomly selected. From those, to evaluate the percentage of offspring that would normally hatch from eggs, 100 eggs were selected for each replication—all eggs laid on the same day by the parent adults Hatching rate was estimated using the following formula:

Hatching rate(%)=(the number of hatching eggs/the total number of eggs)×100.

In the life table study, the intrinsic rate of increase (*r*), the finite rate of increase (*λ*), the net reproductive rate (*R*_0_), the mean generation time (*T*) are four important parameters. Moreover, some other parameters were calculated. The age-stage specific survival rate (*s*_*xj*_) represents the age (*x*) and the stage (*j*) to which each egg can survive. The curve of age-specific survival rate (*l*_*x*_) is a simplified form of the age-stage survival rate (*s*_*xj*_). Age-stage specific fecundity (*f*_*xj*_) means the number of offspring produced by a female of age *x* and stage *j*, because only female adults produce eggs, there is only a single curve *f*_*x*9_ [21]. Age-stage specific reproductive values (*v*_*xj*_) represent the ability of each individual at age *x* and stage *j* to contribute to the future population. The life expectancy (*e*_*xj*_) means the age *x* and stage *j* that each individual is expected to live after age *x*. The preoviposition period (APOP) of female adults was the duration from adult emergence to initial oviposition, and the total preoviposition period (TPOP) of females was calculated from egg to first oviposition [19].

### Effect of cyantraniliprole on feeding indices of fourth-instar larvae of *A*. *ipsilon*

To assess the effect of sublethal cyantraniliprole on the feeding indices of *A*. *ipsilon*, artificial diets containing cyantraniliprole at dose levels of LC_5_, LC_20_ and LC_40_ were prepared and cut into similar-size cubes (approximately 1.5 g per cube). The control was given an artificial diet treated with distilled water. In this experiment, 120 newly molted active fourth-instar larvae (within 24 hours) were selected per treatment and divided among five replications. All the larvae were starved for 4 h before the experiment and then fed with either a treated or untreated (control) artificial diet. Moreover, 50 extra newly molted active fourth-instar larvae (within 24 hours) were weighed, dried in an oven (at 45°C) for 72 h, and then reweighed. The initial dry weight of each larva in the experiment was converted to a fresh-to-dry (fresh: dry) weight ratio. A similar method was used to determine the initial dry weight of the artificial diet cubes used in the various treatment groups. After 72 h, the larvae and any remaining artificial diet and feces produced by the larvae were removed, dried in an oven (at 45°C) for 72 h, and then weighed. Thereby, the dry weight of food eaten (E), the dry weight of feces produced (F) and the dry weight gain of the insects (P) were obtained. All weights were measured using a sensitive balance accurate to (Sartorius BSA 124 S, Max×120 g d = 0.1 mg).

This experiment was continued for three days. The feeding indices were estimated using the following formulae [22]:

Relative consumption rate(RCR)=E/TA,

Relative growth rate(RGR)=P/TA,

Approximate digestibility(AD)=100(E-F)/E,

Efficiency of conversion of ingested food(ECI)=100P/E,

Efficiency of conversion of digested food(ECD)=100P/(E-F).

In the preceding formulae, A = mean of dry weight of larvae during T, E = dry weight of food eaten, F = dry weight of feces produced, P = dry weight gain of insect, T = duration of experimental period.

### Measurement of nutrient contents

Levels of several energy substances were determined in the experiment, including total protein, lipid and carbohydrate. This experiment involved four treatments (LC_5_, LC_20_, LC_40_, and control group) with 200 fourth-instar *A*. *ipsilon* larvae in each treatment. Additionally, 25 newly molted active fourth-instar larvae (within 24 hours) were selected and rinsed with deionized water at times of 6 h, 24 h, 48 h and 72 h after treatment, respectively. All the samples were frozen with liquid nitrogen and stored at -80°C in a freezer. In each biochemical index experiment, one larva was used for each replicate and four replicates were performed for each treatment

Total protein content was determined according to the method described by Bradford [18]. First, one larva was homogenized in 100 μL of 50 mM Tris-HCl (pH = 7.1) containing 0.5% Triton X-100 and 20% sucrose. The sample was then centrifuged at 12,000 rpm for 10 minutes at 4°C. Then, 30 μL of the supernatant was mixed with 150 μL 0.01% Coomassie Brilliant Blue G-250 (Solarbio, Beijing, China) for 5 minutes. Bovine serum albumin (Solarbio) was used to establish the protein standard curve. The absorbance was measured at 595 nm using a BioTek Synergy™ 2 Multi-Mode Reader (BioTek Instruments, Inc. Winooski, Vermont, USA).

The lipid was determined using the method developed by Zhao et al [23]. Each larva was homogenized with 200 μL 2% Na_2_SO_4_ solution individually, and then a mixture of 750μL Chloroform: methanol (2:1) was added so that the lipids could be extracted. Then, all the tubes were centrifuged at 12,000 rpm for 10 minutes (4°C). The supernatant was taken out and dried at 40°C. Then, the samples were dissolved with 500μL H_2_SO_4_ and incubated (90°C) for 10 minutes. Finally, 30 μL of each sample and 270 μL vanillin (Aladdin Industrial Inc., Shanghai, China) (600 mg vanillin dissolved in 100 mL distilled water and 400 mL 85% H_3_PO_4_) were added into an Elisa plate. The absorbance was read at 530 nm after 30 minutes. Total lipid was quantified under the standard curve for cholesterol.

Carbohydrates were determined using the method described by Roe [24]. Each individual larva was homogenized with 300 μL 10% trichloroacetic acid (TCA) solution and then centrifuged at 12,000 rpm for 10 minutes (4°C). Next, 30 μL supernatant was taken out and then added to 70 μL 10% TCA and 600 μL of 0.2% anthrone (200 mg of anthrone dissolved in 100 mL H_2_SO_4_). All the sample tubes were placed in a 90°C water bath for 10 minutes. The absorbance was read at 630 nm and glucose was used to generate the standard curve to calculate the carbohydrate content of each sample.

### Statistical analysis

The data obtained from the toxicity experiment were corrected for control mortality using Abbott’s formula [25] before analysis, and data were analyzed by probit analysis, using the SPSS statistical software (version 18.0, SPSS Inc., Chicago, IL, USA), in order to determine the lethal and sublethal concentrations (LC values). Statistically significant mean values were determined using ANOVA, and the significant differences among the treatments were determined using the Student-Newman-Keuls test (*P*< 0.05).

### Life Table Analysis

In this study, all the data for the life table for *A*. *ipsilon* were analyzed based on the age-stage two-sex life table theory. The developmental time, survivorship and female daily fecundity were calculated using the computer program TWOSEX MS Chart [26].

All the standard errors of the developmental time of each stage, fecundity, longevity of adults and population parameters were estimated by the bootstrap test method [18,27,28]; 200,000 bootstraps were used. Based on the confidence interval of difference, the results of all the different treatments were compared using the paired bootstrap test. Both bootstrap and paired bootstrap test are included in the TWOSEX-MSChart [26], the programs are available without charge and may be downloaded at http://140.120.197.173/Ecology/. All the curves for survival rates, fecundity and reproductive value were constructed using Originpro 8.5.

## Results

### Toxicity of cyantraniliprole to fourth-instar larvae of *A*. *ipsilon*

This experiment determined the toxicity of cyantraniliprole on fourth-instar larvae of *A*. *ipsilon*. After 72 h of treatment the LC_50_ was 0.354 μg.g^−1^ of artificial diet, while the LC_5_, LC_20_, and LC_40_ values were 0.094, 0.179, and 0.289 μg.g^−1^ of artificial diet, respectively (Table 1).

**Table 1:** Toxicity of cyantraniliprole on the fourth instar larvae of *A*. *ipsilon*.

		Concentration μg /g (95% CL)				
Insecticide	N^a^	LC_5_	LC_20_	LC_40_	Slope ± SE	*χ2* (*df*)
Cyantraniliprole	840	0.094 (0.058–0.1277)	0.179 (0.133–0.226)	0.289 (0.229–0.365)	2.848±0.388	1.204 (4)

*χ2* is statistically significant (*p* < 0.05)

N^a^ the number of subjects.

### Sublethal effects of cyantraniliprole on the development and growth of *A*. *ipsilon*

**Fig 1:**
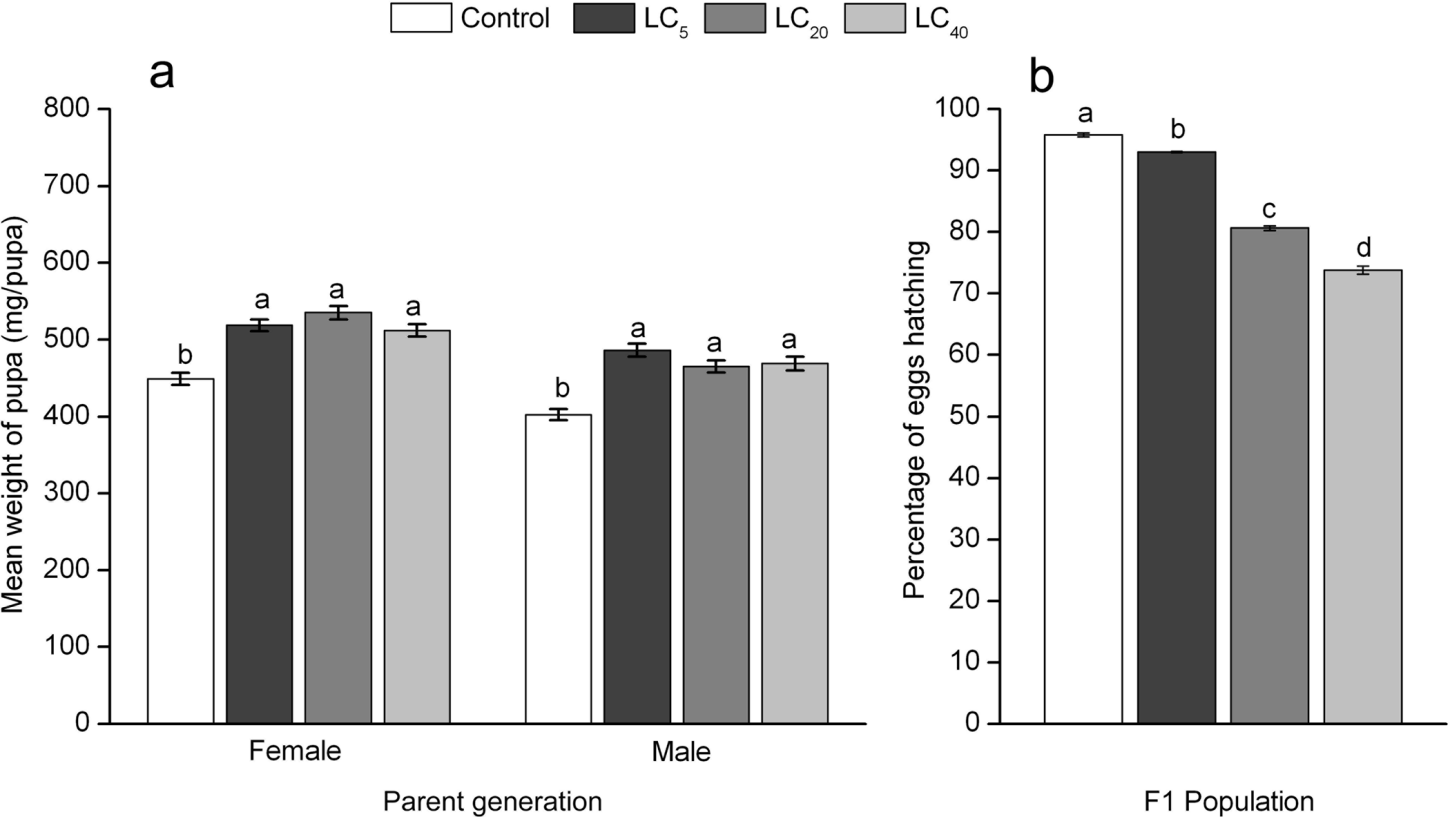
Pupae weight of male and female of *A*. *ipsilon* in parent population (a) and percentage of offspring eggs that hatched in the offspring population (b) after the parent fourth-instar larval stage was exposed to sublethal concentrations of cyantraniliprole. Bars labeled with the same letters do not differ significantly (a, 200,000 bootstraps; b, Student-Newman-Keuls test, *P*< 0.05).

In the parental generation, the sublethal effects of cyantraniliprole on development time, pupa duration and mean egg numbers of female adults of *A*. *ipsilon* were determined (Table 2). Three sublethal concentrations all significantly prolonged the duration of larvae after treated by cyantraniliprole in fourth instar compared with the control. The duration of pupae at LC_5_ (17.63 days), LC_20_ (17.79 days) and LC_40_ (17.67 days) was significantly longer than that of the control (16.37 days), but no significant difference was found between the three treatments. The mean longevity of females and male adults in the LC_5_ (9.544 and 8.670 days), LC_20_ (7.528 and 8.280 days) and LC_40_ (8.395 and 8.000 days) groups was significantly shorter than that of the control group (12.04 and 10.73 days). There were no significant differences between the control and the three treatment groups on adult preovipositional period (APOP). However, compared with the control group, the total preovipositional period (TPOP) of adults in the LC_5_, LC_20_, and LC_40_ treatments was significantly prolonged. The sublethal concentrations of cyantraniliprole had noticeably decreased the mean numbers of eggs laid by female adults. The smallest egg number was found in the LC_40_ group (Table 2). Meanwhile, in the LC_5_, LC_20_, and LC_40_ treated groups, the weights of both female and male pupae were significantly higher than the weights in the control group (Fig 1A). We also found that egg hatchability was significantly lower in the offspring generation after the parent fourth-instar larvae treated with cyantraniliprole (*F* = 607.4, df = 3,16, *P* < 0.0001), and that effect increased along with the increase in concentration (Fig 1B).

**Table 2:** The sublethal effects of cyantraniliprole on the development and growth of *A*. *ipsilon*.

	Control		LC_5_		LC_20_		LC_40_	
Stages	n	Mean ± SE	n	Mean ± SE	n	Mean ± SE	n	Mean ± SE
Egg (day)	120	4.050 ± 0.020 a	120	4.075 ± 0.021 a	120	4.071 ± 0.019 a	120	4.047 ±0.008 a
L1 (day)	120	2.850 ± 0.052 a	120	2.742 ± 0.061 a	120	2.708 ± 0.061 a	120	2.675 ± 0.065a
L2 (day)	120	3.162 ± 0.046 a	120	3.108 ± 0.032 a	120	3.142 ± 0.038 a	120	3.138 ± 0.031 a
L3 (day)	120	3.292 ± 0.044 a	120	3.229 ± 0.036 a	120	3.342 ± 0.052 a	120	3.258 ± 0.036 a
L4 (day)	120	3.762 ± 0.049 c	114	4.272 ± 0.083 b	99	4.611 ± 0.098 a	73	4.487 ± 0.086 ab
L5 (day)	119	4.261 ± 0.080 c	111	5.311 ± 0.115 b	93	5.220 ± 0.132 b	67	5.702 ± 0.223 a
L6—L7(day)	119	9.685 ± 0.117 b	111	11.221 ± 0.186 a	93	11.495 ± 0.190 a	67	11.000 ± 0.239 a
Pupa (day)	108	16.37 ± 0.097 b	104	17.63 ± 0.097 a	79	17.79 ± 0.093 a	66	17.67 ± 0.124 a
Mean longevity of female adult (day)	56	12.04 ± 0.350 a	57	9.544 ± 0.419 b	36	7.528 ± 0.533 c	33	8.395 ± 0.581 bc
Mean longevity of male adult (day)	52	10.73 ± 0.466 a	47	8.670 ± 0.556 b	43	8.280 ± 0.437 b	33	8.000 ± 0.355 b
APOP (day)	56	2.884 ± 0.062 a	54	2.907 ± 0.063 a	32	2.844 ± 0.085 a	29	2.949 ± 0.090 a
TPOP (day)	56	50.71 ± 0.264 b	54	54.20 ± 0.337 a	32	54.59 ± 0.408 a	29	55.19 ± 0.408 a
Mean number of eggs(per female)	56	1638 ± 46.46 a	57	1081 ± 62.03 b	36	712.6 ± 73.65 c	33	499.5 ± 40.44 d

Standard errors were estimated by using 200,000 bootstraps. Means marked with different letters in the same row are significantly different as calculated using the paired bootstrap test at the 5% significance level. APOP, adult preovipositional period; TPOP, total preovipositional period.

### Sublethal effects of cyantraniliprole on the population growth parameters of *A*. *ipsilon*

After treatment with cyantraniliprole, compared to the control group (0.125 d^−1^), the intrinsic rate of increase (*r*) decreased obviously (0.111 d^−1^, 0.094 d^−1^ and 0.086 d^−1^ for LC_5_, LC_20_ and LC_40_, respectively). The tendency of the finite rate of increase (*λ*), the net reproductive rate (*R*_0_) after cyantraniliprole treatment, was similar to the intrinsic rate of increase (*r*); however, the mean generation time (*T*) of individuals in the three treatment groups was significantly prolonged compared with the control group (Table 3).

**Table 3:** The sublethal effects of cyantraniliprole on *A*. *ipsilon* parent population parameters.

	Control	LC_5_	LC_20_	LC_40_
Population parameters	Mean ± SE	Mean ± SE	Mean ± SE	Mean ± SE
Intrinsic rate of increase (*r*) (d^-1^)	0.125 ± 0.002 a	0.111 ± 0.002 b	0.094 ± 0.003 c	0.086 ± 0.003 d
Finite rate of increase (*λ*) (d^-1^)	1.133 ± 0.002 a	1.117 ± 0.002 b	1.099 ± 0.003 c	1.090 ± 0.003 c
Net reproductive rate (*R*_*0*_) (offspring)	764.4 ± 77.55 a	513.9 ± 57.28 b	213.7 ± 36.96 c	137.4 ± 23.12 c
Mean generation time (*T*) (d)	53.241 ± 0.262 b	56.147 ± 0.407 a	56.584 ± 0.526 a	56.824 ± 0.497 a

Standard errors were estimated by using 200,000 bootstraps. Means marked with different letters in the same row are significantly different as calculated using the paired bootstrap test at the 5% significance level.

**Fig 2:**
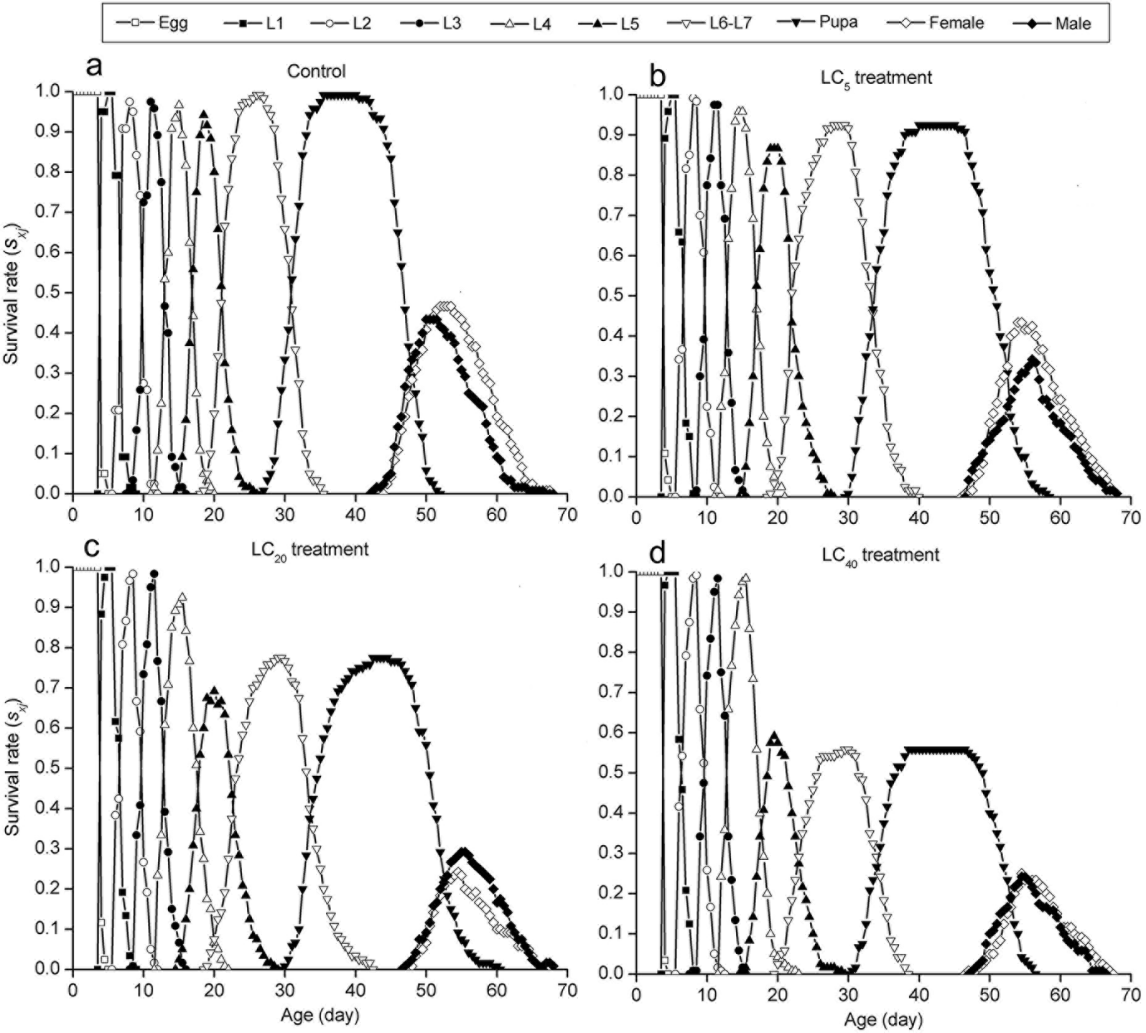
Age-stage specific survival rate (*s*_*xj*_) of *A*. *ipsilon* exposed to sublethal concentrations of cyantraniliprole.

**Fig 3:**
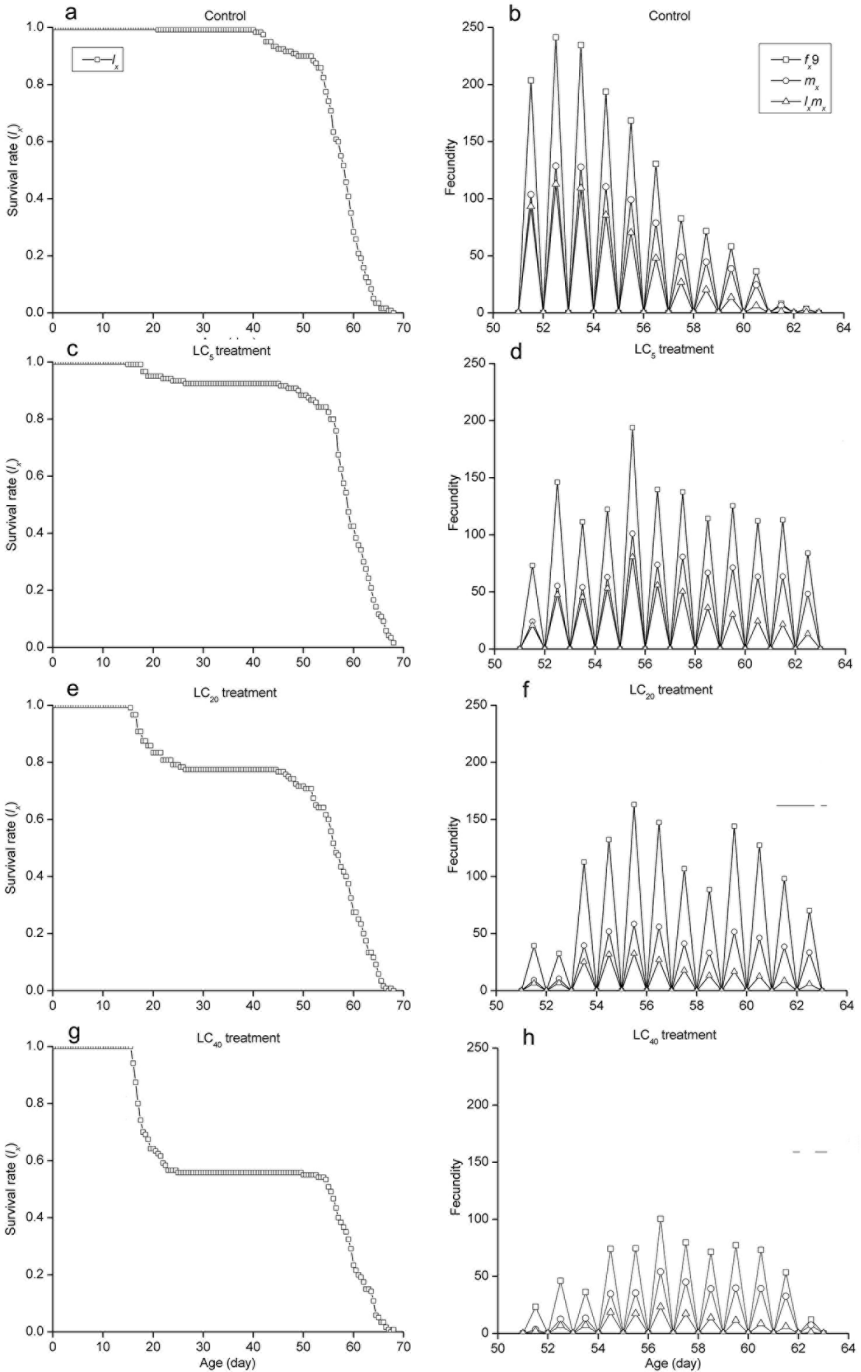
Age-specific survival rate (*l*_*x*_), female age-specific fecundity (*f*_*x*_9), age-specific fecundity of the total population (*m*_*x*_), and age specific maternity (*l*_*x*_*m*_*x*_) of *A*. *ipsilon* exposed to sublethal concentrations of cyantraniliprole.

**Fig 4:**
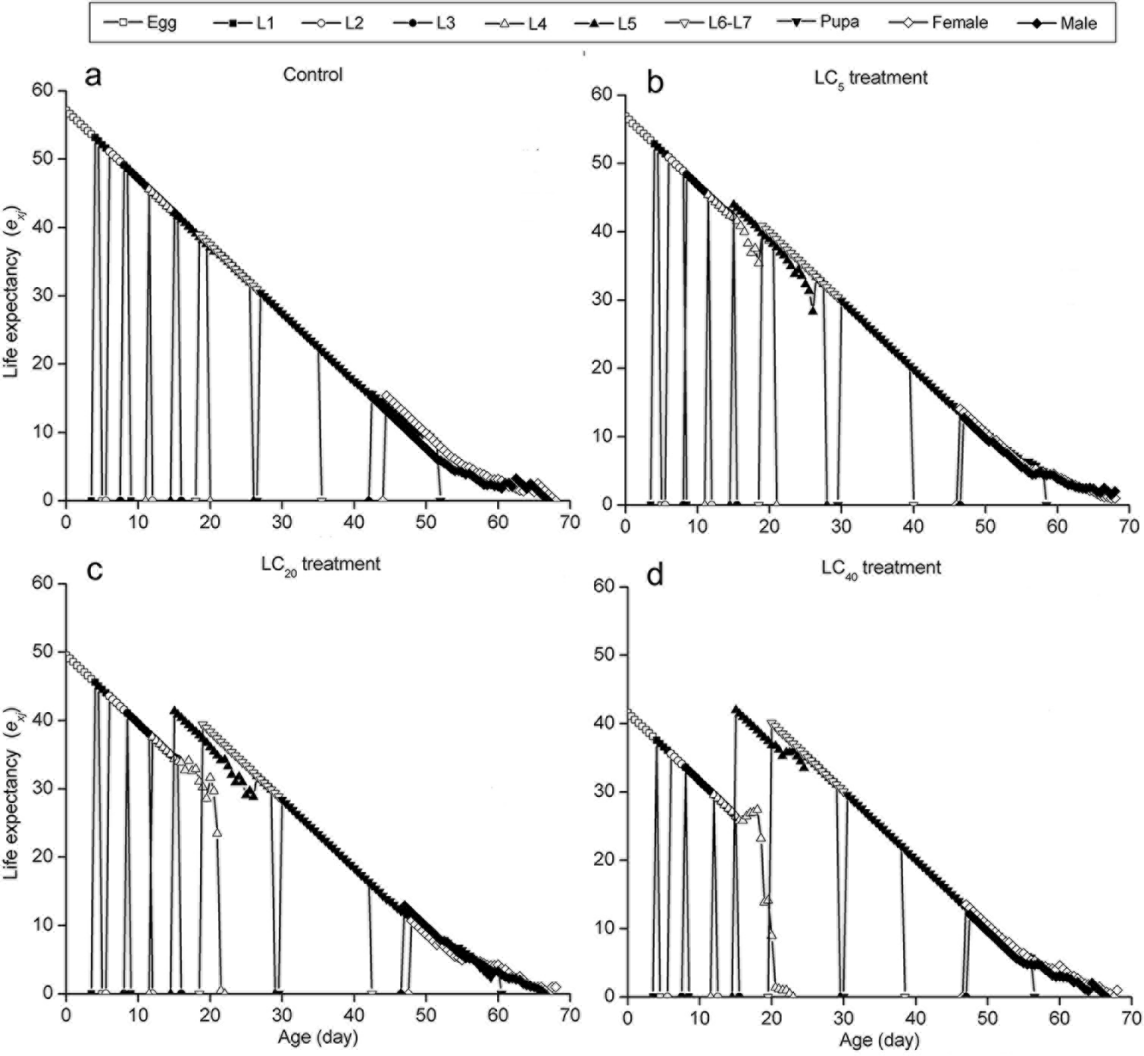
Life expectancy (*e*_*xj*_) of *A*. *ipsilon* exposed to sublethal concentrations of cyantraniliprole.

**Fig 5:**
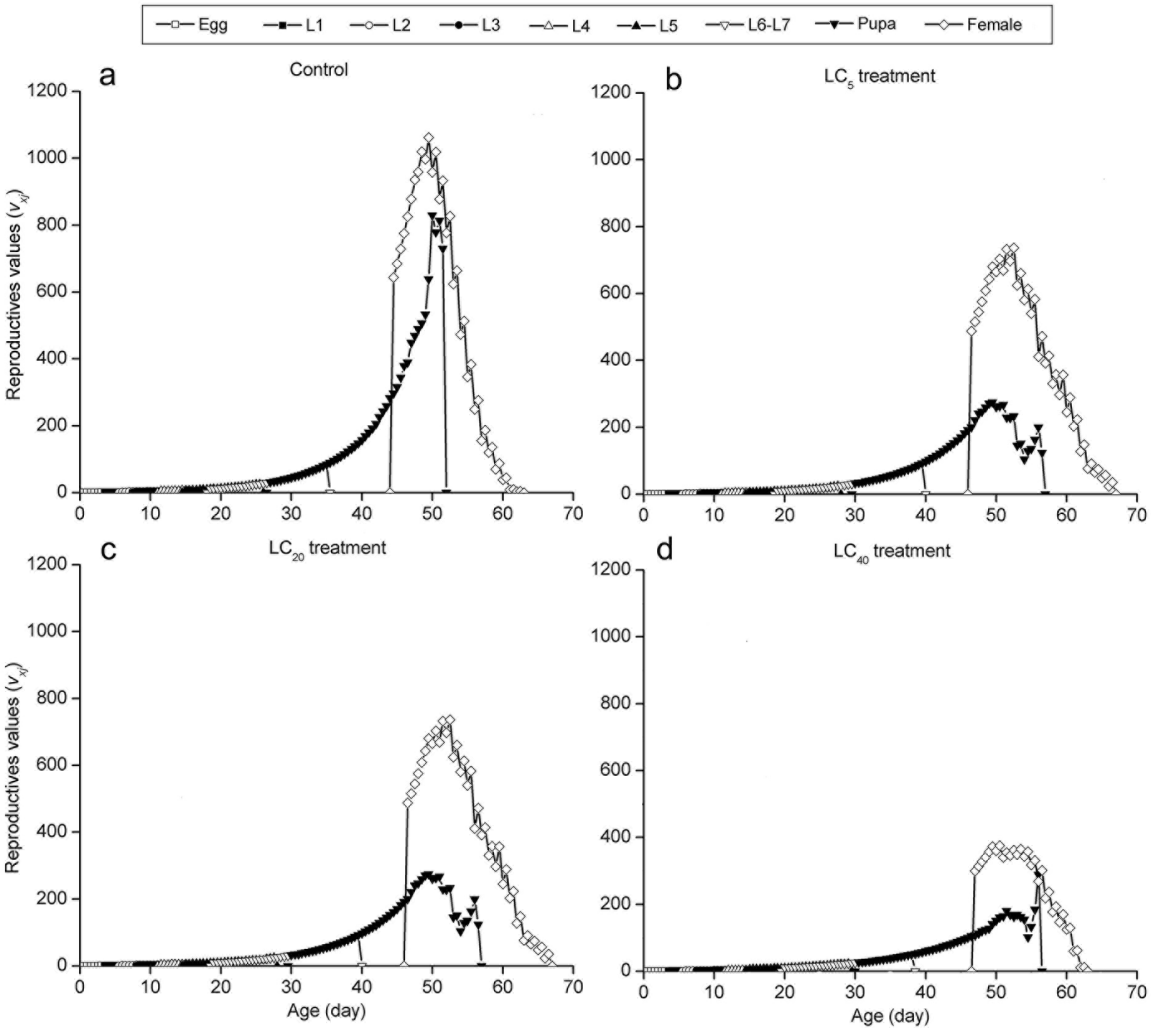
Age-stage specific reproductive values (*v*_*xj*_) of *A*. *ipsilon* exposed to sublethal concentrations of cyantraniliprole.

The curves of the age-stage specific survival rate (*s*_*xj*_) were shown in Fig 2. Observation overlaps were apparent between stages because the development rate varied among individuals. In the control group, 90% of eggs survived to the adult stage. However, the survival rate was significantly lower in the LC_5_ (76.66%), LC_20_ (53.34%) and LC_40_ (49.17%) groups (Fig 2).The curve of age-specific survival rate (*l*_*x*_) was a simplified form of the age-stage survival rate (*s*_*xj*_). The highest peak of age-special fecundity (*m*_*x*_) appeared at 52.54 days in the control group (126.91 eggs). For the LC_5_ (100.69 eggs), LC_20_ (58.66 eggs) and LC_40_ (53.76 eggs) groups, the highest peaks occurred at 55.57, 55.61 and 56.56 days, respectively. The curve of *l*_*x*_*m*_*x*_ was formed on the basis of *f*_*x*9_ and *m*_*x*_, meaning the total fecundity of the population (Fig 3). The curves of the life expectancy (*e*_*xj*_) were shown in Fig 4. The *e*_*xj*_ declined sharply at the fourth instar larval stage in the three treated groups but increased significantly for fifth instar larval stage, while the *e*_*xj*_ of eggs declined in the three treated groups compared to the control group (Fig 4). The age-stage specific reproductive values (*v*_*xj*_) curves were shown in Fig 5. The results here showed that the highest peaks of *v*_*xj*_ at the pupal stage declined sharply, while the time required reaching the highest peaks were prolonged in the LC_5_, LC_20_ and LC_40_ groups compared with the control group. Similar effects were found at the female adult stage (Fig 5).

### Sublethal effects of cyantraniliprole on feeding indices of *A*. *ipsilon*

The feeding indices of *A*. *ipsilon* fourth-instar larvae were seriously affected when they were fed on the artificial diet mixed with cyantraniliprole. A sublethal dose of cyantraniliprole greatly affected both *A*. *ipsilon* feeding behavior and growth. The approximate digestibility (AD) for the LC_5_, LC_20_ and LC_40_ groups was significantly increased compared with the control group (*F* = 126.6, df = 3,16, *P* < 0.0001); however, the efficiency of food ingestion (ECI) (*F* = 54.43, df = 3,16, *P* < 0.0001), efficiency of food digestion (ECD) (*F* = 101.5, df = 3,16, *P* < 0.0001), relative growth rate (RGR) (*F* = 2229, df = 3,16, *P* < 0.0001) and relative consumption rate (RCR) (*F* = 422.3, df = 3,16, *P* < 0.0001) were significantly lower. These effects were intensified at higher concentrations. Significant differences were found between three treatments (Table 4).

**Table 4:** Feeding indices of fourth instar larvae of *A*. *ipsilon* after treatment with cyantraniliprole.

Treatments	AD (%)	ECI (%)	ECD (%)	RCR (mg/mg/day)	RGR (mg/mg/day)
Control	69.07 ± 0.870 c	21.64 ± 0.553 a	31.37 ± 1.0454 a	6.55 ± 0.244 a	1.41 ± 0.022 a
LC_5_	82.47 ± 0.690 b	15.34 ± 0.444 b	18.60 ± 0.5337 b	1.85 ± 0.047 b	0.28 ± 0.010 b
LC_20_	83.85 ± 0.435 ab	13.79 ± 0.295 bc	16.44 ± 0.2834 bc	1.31 ± 0.032 c	0.18 ± 0.005 c
LC_40_	85.82 ± 0.639 a	12.71 ± 0.764 c	14.82 ± 0.8826 c	0.89 ± 0.053 d	0.11 ± 0.008 d

Means ± standard deviation followed by the same letter within columns indicate no significant difference (Student-Newman-Keuls test: P < 0.05). RGR—relative growth rate, RCR—relative consumption rate, ECI—efficiency of food ingestion, ECD—efficiency of food digestion, AD—Approximate digestibility.

### Effects of cyantraniliprole on the amount of nutrient contents of *A*. *ipsilon*

**Fig 6:**
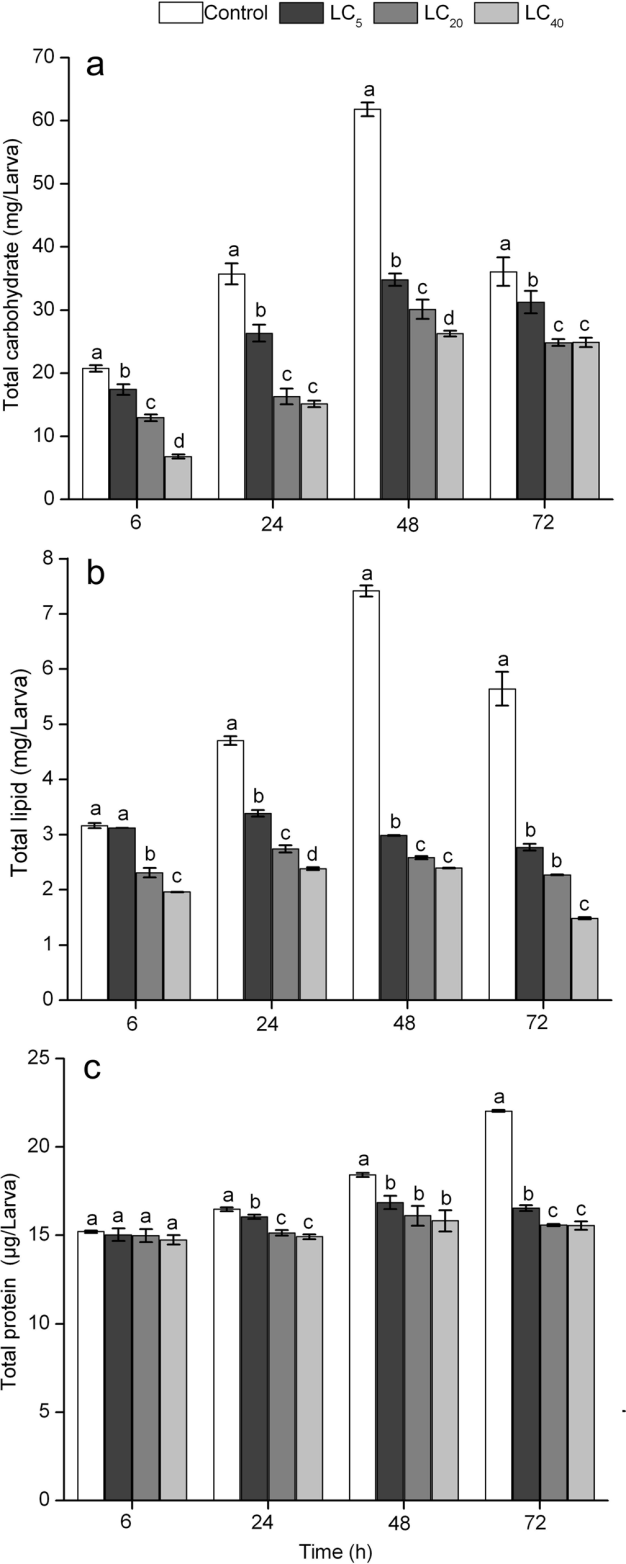
The amount of nutrients (a: carbohydrate, b: lipid, c: total protein) in fourth-instar larvae of *A*. *ipsilon* (Mean ± SE) after treatment with sublethal concentrations of cyantraniliprole. Bars labeled with the same letters do not differ significantly (Student-Newman-Keuls test, *P*< 0.05).

The present study showed that cyantraniliprole had a great influence on the amount of major energy substances such as carbohydrate, lipid and protein found in fourth-instar larvae of *A*. *ipsilon*. The amount of total carbohydrate in larvae in the LC_5_, LC_20_, and LC_40_ cyantraniliprole groups declined intensely compared with the control group (6h: *F* = 106.9, df = 3,12, *P* < 0.0001; 24h: *F* = 58.73, df = 3,12, *P* < 0.0001; 48h: *F* = 221.3, df = 3,12, *P* < 0.0001; 72h: *F* = 13.10, df = 3,12, *P* < 0.0001) (Fig 6A). Similarly, from 24 h to 72 h, the lipid content of larvae in the LC_5_, LC_20_, and LC_40_ treatments was significantly lower compared with the control (6h: *F* = 246.4, df = 3,12, *P* < 0.0001; 24h: *F* = 167.6, df = 3,12, *P* < 0.0001; 48h: *F* = 2321, df = 3,12, *P* < 0.0001; 72h: *F* = 227.3, df = 3,12, *P* < 0.0001); however, no significant difference was found between the LC_5_ group and the control group at 6 h (Fig 6B). In both control and cyantraniliprole-treated groups, the amount of carbohydrate and lipids increased from 6 h to 48 h after treatment; then declined at 72 h. The amount of protein in the treated larvae was not significantly different at 6 h compared to the control (6h: *F* = 0.447, df = 3,12, *P* = 0.724); however, it significantly decreased at 24, 48, and 72 h after cyantraniliprole treatment (24h: *F* = 27.30, df = 3,12, *P* < 0.0001; 48h: *F* = 6.365, df = 3,12, *P* = 0.008; 72h: *F* = 441.4, df = 3,12, *P* < 0.0001) (Fig 6C).

## Discussion

The present study showed that cyantraniliprole caused high mortality in *A*. *ipsilon*; the LC_50_ value occurred after consumption of 0.354 μg.g^-1^ of artificial diet. The sublethal effects on larval development may be due to perturbations in the development of neural tissues by neurotoxic substances [29]. In the present study, both larval duration and pupal stage were prolonged after exposure to sublethal concentrations of cyantraniliprole. Another anthranilic diamide insecticide, chlorantraniliprole, also prolonged larval duration and the pupal stages of lepidopteran pests such as *Ostrinia furnacalis*, *Plutella xylostella* and *Spodoptera exigua* when exposed at sublethal concentrations [15,30,31]. Because the cyantraniliprole-treated *A*. *ipsilon* larvae must spend more resources on detoxification rather than development, larval development takes significantly longer than in the control group [32]. Several reports indicated that the mean number of eggs laid by female adults and the percentage of offspring eggs hatching would decrease after treatment of anthranilic diamide insecticides [7,15,31,33,34].

Reductions in fecundity after treated with pesticides may be result from both physiological and behavioral effects [29]. In our results, both adult longevity and mean number of eggs laid by female adults and percentage of eggs hatching of *A*. *ipsilon* declined after exposure to sublethal doses of cyantraniliprole. This may be due to the fact that the ovaries of female adults were affected by cyantraniliprole, which caused a reduction in egg laying. Perveen et al. [35] showed that LC_30_-chlorfluazuron could reduce the length of different parts of the ovarioles, the number of mature ova, the size of basal oocytes and thickness of their follicular epithelium of *Spodoptera litura*, which reasonable for the reduction in fecundity and hatchability. Previous studies showed that anthranilic diamide insecticide had a significant disruptive effect on both mating behavior and ovaries of Lepidoptera pests [36,37]. We also found that pupal weight was increased after treatment with sublethal cyantraniliprole. This result was in line with Lai et al who found that the pupa of *S*. *exigua* were heavier in LC_30_-chlorantraniliprole-treated groups than in controls [33]. Additionally, Yu et al. [7] and Song et al.[8] reported that pupa weight of *S*. *exigua* and *S*. *litura* increased in cyantraniliprole-treated groups. This phenomenon may be explained as follows [33]: (1) cyantraniliprole killed weaker individuals outright, while the remaining individuals were simply stronger and more robust; and (2) cyantraniliprole increased the duration of larval instars and slowed metamorphosis from larvae to pupae. A higher percentage of supernumerary instars larvae (seven-instar larvae) was found in the cyantraniliprole-treated group, these two reasons both contributed to the appearance of heavy pupae in the results. Additionally, the egg hatching percentage of *A*. *ipsilon* declined significantly in groups treated with cyantraniliprole. In sum, reducing the number of eggs laid by female adults in the parental generation and reducing the egg hatching percentage are both important factors for success in pest management.

The life table study has been recommended as a comprehensive method for evaluating the total effect of an insecticide on an insect population [18]. Age-stage, two-sex life table studies provide a valuable tool for assessing the effects of cyantraniliprole on *A*. *ipsilon* populations using survival rate, adult longevity, fecundity and population parameters. In this study, the age-stage specific survival rate (*s*_*xj*_), fecundity (*m*_*x*_), reproductive values (*v*_*xj*_) and life expectancy (*e*_*xj*_) decreased dramatically in three treated groups. The *e*_*xj*_ declined sharply at the stage of fourth-instar in the three treated groups and significantly increased from the fifth-instar onward because cyantraniliprole kills the weakest individuals in fourth-instar, whereas stronger individuals exhibited a higher tolerance to cyantraniliprole and subsequently survived better into the subsequent stages. Meanwhile, the *e*_*xj*_ of eggs was lower in the three treated groups compared to the control group because of the increasing pressure of cyantraniliprole. Additionally, the intrinsic rate of increase (*r*), the finite rate of increase (*λ*), and the net reproductive rate (*R*_0_) tended to be lower in the cyantraniliprole-treated groups than in the control groups. Han et al. [15] reported that sublethal doses of chlorantraniliprole had similar results on *P*. *xylostella*. Therefore, all these parameters indicated that sublethal doses of cyantraniliprole on *A*. *ipsilon* can affect population dynamics via reductions in survival and reproduction capacity.

Insecticide hormoligosis may result in pest resurgence and secondary outbreaks [38,39]. In insecticidal hormoligosis, certain sublethal doses of insecticides increase fecundity and population parameters of insects. Hormoligosis has been shown in some insects. Yin et al.[40] reported that population growth of *P*. *xylostella* was enhanced after exposure to sublethal concentrations (LC_25_) of spinosad. Tang et al. [16] also reported that lower concentrations of sulfoxaflor showed a hormesis-induced effect on *Myzus persicae*. Adverse behavior on insects caused by sublethal insecticides may due to the species and physiological states of insects and to differences in the classes and concentrations of the insecticides used [15,41]. In this study, hormoligosis was not observed in cyantraniliprole-treated groups based on lower survival rate, numbers of offspring eggs laid by the parent and population parameters of *A*. *ipsilon* when sublethal concentrations of cyantraniliprole were applied to fourth-instar larvae in the parent generation. Similar conclusions were obtained for the offspring generation (F1) (Xu et al. results unpublished). Future studies need to focus on the efficacy of sublethal concentrations of cyantraniliprole on successive generations of *A*. *ipsilon*.

In the present study, development at the larval and pupal stages was prolonged significantly. This may be because cyantraniliprole prevents insects from feeding, leading to insufficient nutrients for normal insect growth [32]. To determine this, we evaluated the differences between feeding indices in the control and treatment groups. The results showed that the approximate digestibility (AD) of *A*. *ipsilon* was increased in larvae fed with cyantraniliprole-treated artificial diets. Most probably, the treated larvae attempt to overcome and compensate for this drawback of reducing consumption. The result agreed with the opinion of Nathan [42], in which longer larva duration and the persistence of food in the digestive tract resulted in higher approximate digestibility. Stoyenoff et al indicated that enhanced digestibility was due to the food passing through the digestive tract more slowly when the target insect had eaten less [43]. The relative growth rate (RGR), an index of growth coupled with the weight of the insect larvae decreased greatly in cyantraniliprole-treated *A*. *ipsilon* larvae. This result agreed with Senthil-Nathan that the RCI, RGR, ECI and ECD of *Cnaphalocrocis medinalis* fourth-instar larvae was significantly reduced after treatment with Melia azedarach [42], or biopesticides [44]. Liu et al. [45] also reported that fraxinellone could reduce the RGR of *O*. *furnacalis* (Guenée). Jansen and Groot [46] indicated that the reduced RGR might have resulted from irreparable damage to the cellular surface of the midgut lumen. The RGR decreased sharply with increased concentration of insecticides, indicating that the food was not suitable for the insect and may act as an inhibitor, so that the treated larvae did not have sufficient specific components for normal growth and metabolism processes to occur [47]. In conclusion, the lower RCI, RGR, ECI and ECD values as well as the higher AD showed that larvae treated with cyantraniliprole faced lower feeding indices. This might be because more food resources had to be used for detoxification, leaving insufficient essential components for normal growth, and may have a direct relationship with the developmental delay, low survival rate and reproduction of treated *A*. *ipsilon* populations.

In an insect’s body, proteins, lipids and carbohydrates are three required chemical substances closely linked with some important metabolic processes. The amounts of these three substances vary among different growth stages and feeding conditions [48]. Many insecticides reduce feeding efficiency—which in turn reduces these biochemical components in the body [23,49]. In the present study, total protein, lipids, and carbohydrates in the fourth-instar larvae of *A*. *ipsilon* were reduced after treatment with cyantraniliprole. This result was in line with Zhao et al. [23] that proteins, lipids and carbohydrates in fourth-instar larvae of *Bradysia odoriphaga* were reduced after being treated with benzothiazole. Etebari et al. [50] suggested that total protein in silkworm larva was greatly reduced at 72 h after treatment with pyriproxyfen. Additionally, Nath et al. [51,52] reported organophosphorus insecticides under sublethal concentrations could decrease the total proteins and carbohydrates in silkworm instar larvae. The present result may be because cyantraniliprole caused protein to be degraded into amino acids to take part in the TCA cycle of acetic acid and altered carbohydrate metabolic functions to make up for the lower energy under the stress of insecticides in the body [51,52]. In addition, detoxification in the larvae required a larger portion of consumed substances to be transformed into energy after cyantraniliprole treatment, which may be another reason for the reduction in the content of proteins, lipids and carbohydrates in *A*. *ipsilon* larvae.

In sum, this study showed that a sublethal dose of cyantraniliprole prolongs the duration of larval and pupal stages and decreases survivorship, longevity and fecundity of *A*. *ipsilon* adults. Finally, it slows the speed of population growth of *A*. *ipsilon*, and no hormoligosis phenomenon appeared during testing. These results provide useful information for control strategies targeting *A*. *ipsilon*. In addition to directly suppressing the population density of *A*. *ipsilon* using lethal doses of cyantraniliprole, some larvae would contact with sublethal doses of this insecticide after application in the field. Our results suggested that sublethal doses of cyantraniliprole could prolong *A*. *ipsilon* developmental rates, limit its survival rate and reduce its reproduction capacity, thus controlling population density effectively. This strategy could reduce both application frequency and total amount of pesticides used, which would aid in reducing both control costs and environmental pollution. These unique advantages suggest that cyantraniliprole has vast application prospects in crop production.

However, this study only investigated the effects of three sublethal concentrations of cyantraniliprole on *A*. *ipsilon*, further studies are needed to investigate wider range of sublethal concentrations on this pest, including those below no observed effect concentration (NOEC) to check if hormesis occur in *A*. *ipsilon*. Moreover, in order to obtain comprehensive information on the application of cyantraniliprole on *A*. *ipsilon*, the study of a possible genetic variability in response to sublethal doses should also be carried out.
